# Portable device for the detection of colorimetric assays

**DOI:** 10.1098/rsos.171025

**Published:** 2017-11-01

**Authors:** G. S. Luka, E. Nowak, J. Kawchuk, M. Hoorfar, H. Najjaran

**Affiliations:** School of Engineering, University of British Columbia, 333 University Way, Kelowna, British Columbia, Canada V1V1V7

**Keywords:** colorimetric detection, portable device, point of care, sensors, nitrite detection, pH detection

## Abstract

In this work, a low-cost, portable device is developed to detect colorimetric assays for in-field and point-of-care (POC) analysis. The device can rapidly detect both pH values and nitrite concentrations of five different samples, simultaneously. After mixing samples with specific reagents, a high-resolution digital camera collects a picture of the sample, and a single-board computer processes the image in real time to identify the hue–saturation–value coordinates of the image. An internal light source reduces the effect of any ambient light so the device can accurately determine the corresponding pH values or nitrite concentrations. The device was purposefully designed to be low-cost, yet versatile, and the accuracy of the results have been compared to those from a conventional method. The results obtained for pH values have a mean standard deviation of 0.03 and a correlation coefficient *R*^2^ of 0.998. The detection of nitrites is between concentrations of 0.4–1.6 mg l^−1^, with a low detection limit of 0.2 mg l^−1^, and has a mean standard deviation of 0.073 and an *R*^2^ value of 0.999. The results represent great potential of the proposed portable device as an excellent analytical tool for POC colorimetric analysis and offer broad accessibility in resource-limited settings.

## 1. Introduction

Recently, there has been a growing demand to monitor and control all aspects of our urban environment in real time. This is brought about by increasing concerns with environmental, health, security and safety issues. Hence, there is a need to develop low-cost, versatile and portable diagnostic tools for early detection of harmful contaminants readily and accurately in remote areas [1,2]. Many analytical tools have been used in the field of medical diagnostics, environmental applications, food analysis, and security and defence. These tools rely on polymerase chain reaction (PCR) [3–5], high-performance liquid chromatography (HPLC) [6], mass spectroscopy [7,8] and enzyme-linked immunosorbent assay (ELISA) [9,10] for analysis. These standard methods for the detection of specific targets possess high accuracy and sensitivity. However, they are expensive, bulky, time-consuming, complicated and need technological improvement, and require trained personnel, which together hinder their applicability for point-of-care (POC) applications [1,11–13].

Advances in digital imaging technology allow for the implementation of sensitive detection and processing methods using cost-effective and high-resolution cameras for colorimetric analysis. It is now possible to exchange results of POC tests in remote areas with subject matter experts without delay by using smartphones [14]. More recently, cloud computing [14] has provided a remarkable promise towards bioanalytical needs, mobile environmental, medical diagnostics and the development of POC testing. Cloud computing enables immediate data transmission and access to information for food, environmental and medical analysis centres worldwide via wireless connections [15], especially in resource-limited settings and remote areas, enabling intervention when required [16].

Colorimetric methods have proved to be fast, adaptable, cost-effective and sensitive for the detection of a wide range of different analytes [17–20] (in a single test) [21,22] ranging from toxic chemicals [23–25] to pathogens [26–28], which can be detected using either the naked eye or optical instruments. However, they are limited to laboratory-controlled settings [29] due to the complexity and fragility of the instruments used for detection. They also need well-trained personnel to run the instruments, which may hinder early diagnostics in remote areas [30–32]. As a result, the integration of these colorimetric assays into portable, versatile, cost-effective, fast and reliable optical digital detectors with onboard processing abilities will: (i) increase the impact and usage of these sensing methods, (ii) reduce time and cost of such tests [33], (iii) provide reliable and rapid analysis and (iv) provide the means for POC diagnostic tools [34,35].

Here, we report the development of a low-cost portable diagnostic device for rapid pH determination and nitrite concentration detection (as a proof of concept) to detect colorimetric assays for in-field and POC diagnostics. Nitrite was chosen as one of the analytes to be detected because of the major worldwide concern about its pollution in water. Nitrite is widely used as a preservative and additive in food production, bleaches and dyes in the industry, and fertilizer in agriculture. However, it is classified as a health hazard because high nitrite concentration in water can cause many diseases such as methaemoglobinaemia [36], miscarriages and central nervous system defects at birth [37].

**Figure 1.**
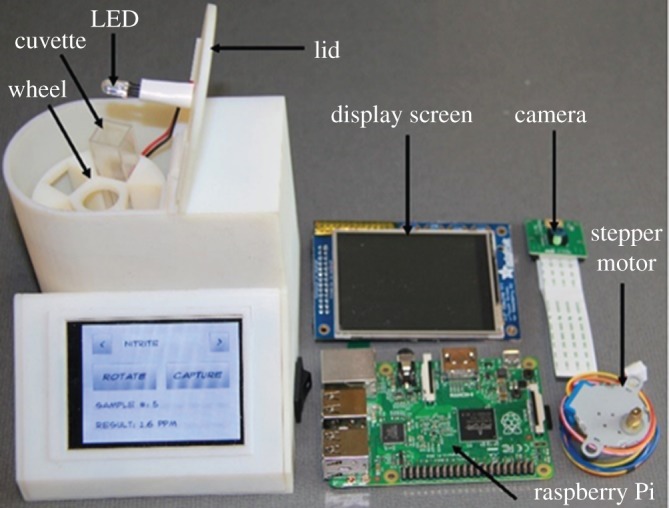
The portable colorimetric device and its major components.

The developed device is battery-powered, wirelessly connected and equipped with a low-cost, single-board computer (Raspberry Pi) for data processing, storage and exchange. A light-emitting diode (LED) provides backlighting, and an 8-megapixel resolution digital camera is used to image and record the colour change in five different cuvettes containing the specific reagents required to perform the colorimetric assay. A rotating carousel sitting on an electromotor is used to position the cuvettes automatically in front of the camera consecutively (figure 1). To enable fast prototyping, the device and its components were fabricated using three-dimensional (3D) printing technology. The use of a stable and reliable external light source and a fixed distance between the sample and the camera in the designed device has allowed the device to be used in different light conditions. Also, it has decreased the interference from ambient light and improved measurements reproducibility. These two drawbacks significantly limit the use of smartphones in sensing technologies and for POC applications [38,39]. Using a standalone device rather than a smartphone also reduces the potential setbacks caused by new models of phones in the future and any incompatibilities that may arise [40,41].

The device also incorporates onboard electronics that calculate saturation value of the (hue–saturation–value, HSV) coordinates of the recorded images. The presence of nitrite or change in the pH value of the sample causes a detectable colour change in the sample cuvette. The cuvette is then placed in one of the five slots of a rotating carousel. An image of the assay is taken with the integrated camera and digitally processed using the single-board computer in real time. Hue and saturation coordinates of the HSV colour space are used to determine the pH and nitrite concentration in the sample, respectively. The image-processing algorithm used decreases the effect of ambient light when the picture is taken and adjusts the cuvette's position in the image frame. Compared with images taken by smartphones for the detection of colorimetric assays [42], the use of a fixed camera in the proposed portable device (as demonstrated in this work) reduces the likelihood of human error, which can compromise repeatability, sensitivity and reliability of colorimetric assays for personal use and POC testing.

## 2. Material and methods

### 2.1. Colorimetric assay preparation

For the preparation of the pH-sensitive colorimetric assay, bromothymol blue (CAS no. 76-59-5), methyl red (CAS no. 493-52-7), sodium hydroxide (CAS no. 1310-73-2), ethyl alcohol (ethanol) (CAS no. 64-17-5), sodium phosphate monobasic monohydrate (CAS no. 10049-21-5) and sodium phosphate dibasic (CAS no. 7558-79-4) were purchased from Sigma-Aldrich (Canada). For the preparation of the nitrite colorimetric assay, *N*-1-naphthylethylenediamine dihydrochloride (NED) (CAS no. 1465-25-4), sulfanilamide (CAS no. 63-74-1) and sodium nitrite (CAS no. 7632-00-0) were purchased from Sigma (Sigma-Aldrich, Canada).

### 2.2. Portable device fabrication

This section outlines the fabrication and construction of the electronic and hardware components of the portable colorimetric device (figure 1). Table 1 outlines the major hardware components and their costs.

**Table 1.** Major hardware components and their costs.

part	cost (USD)
(i) Raspberry Pi Model B	$45
(ii) Raspberry Pi Camera Module	$30
(iii) 2.8′′ PiTFT Resistive Touchscreen 1.5	$35
(iv) VeroWhite 3D Printing Material	$150
total	$260

The electronic hardware components used to construct the device are a Raspberry Pi computer (i), Raspberry Pi camera module (ii) and 2.8′′ PiTFT touchscreen (iii). These are used to collect image samples, process the collected images and display the nitrite concentration and pH values to the user. The Raspberry Pi Model B single-board computer was selected as the embedded controller for the device due to its low-cost, small size (the size of a credit card) and availability of open source support libraries. Power leads were soldered to test points 1 and 2 (TP1 and TP2) on the Raspberry Pi board to supply 5 V power directly to the board, bypassing the USB connector and onboard power regulators. Power is supplied to the onboard electronics by a 3.7 V 2500 mAh Lithium polymer battery. The battery is connected to a PowerBoost 500 C charging circuit from Adafruit Industries. This allows the device to be charged using a 5 V micro-USB connector and boosts the 3.7 V output of the battery to 5.2 V required by the Raspberry Pi and stepper motor. This option allows the portable device to operate on battery power for several hours and allows for simple charging using a standard micro-USB phone charger.

The device can be controlled by a user-friendly touchscreen interface to simplify operation. The 2.8′′ resistive PiTFT touchscreen display is mounted at an angle on the front of the device; a resistive display was chosen over a capacitive screen to allow the operator to control the device while wearing gloves. Samples are loaded into rotating cuvettes, and a 5 V geared stepper motor positions the samples in front of the camera. A ULN2803 8-Channel Darlington Driver IC is used to control the stepper motor and activate a white super bright LED from the Raspberry Pi's general purpose input output (GPIO) pins when capturing images. The LED was placed in the centre of the rotating carousel, allowing it to backlight the cuvette being imaged.

The stock Raspberry Pi camera lens was modified to reduce the working distance to 15 mm by unscrewing the lens to its outermost thread. The device was outfitted with a RTL8188eu Wi-Fi chip enabling connection to a 2.4 GHz wireless network to transfer data for external storage or further analysis on an external computer. A three-dimensional-printed enclosure was designed to house all components inside a compact unit.

### 2.3. pH and nitrite colorimetric assay preparation

**Figure 2.**
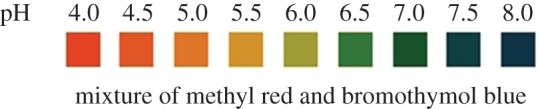
A sample of colour values for the mixed pH indicator.

The colorimetric assay for the detection of pH was prepared to cover the required pH range from 4 to 8. Two different acid–base indicators: methyl red ranging (from 4 to 6) and bromothymol blue ranging (from 6 to 8) were used for the detection of pH. The pH range was selected based on the acceptable range for drinking water in most worldwide regulations [43]. A mixture of the two indicators was prepared to measure a wider pH scale (from 4 to 8) in one sample (figure 2). To measure the pH value, the mixture of two pH indicators (double indicators) was used. The first indicator was prepared by dissolving 0.02** **g of methyl red in 60** **ml of ethyl alcohol. Then, DI water (with a resistivity of 18.2 MΩ using the Millipore Synergy water purification system) was added to increase the volume of the solution to 40** **ml. For the second indicator, 0.1** **g of bromothymol blue was dissolved in 16.0** **ml of NaOH (0.01 M). Then, the solution was diluted to 250** **ml with DI water. For the pH colorimetric assay, 250 µl of bromothymol blue and 50 µl of methyl red were used. For the nitrite concentration determination, two different solutions and four different concentrations of sodium nitrate were prepared: (i) 1% sulfanilamide solution was prepared by dissolving 0.15** **g of sulfanilamide in 15** **ml of 3 M HCl and (ii) 0.02% *N*-1-napthylethylene diamine dihydrochloride solution was prepared by dissolving 2** **mg of *N*-1-napthylethylene diamine dihydrochloride in 100** **ml of DI water.

**Figure 3.**
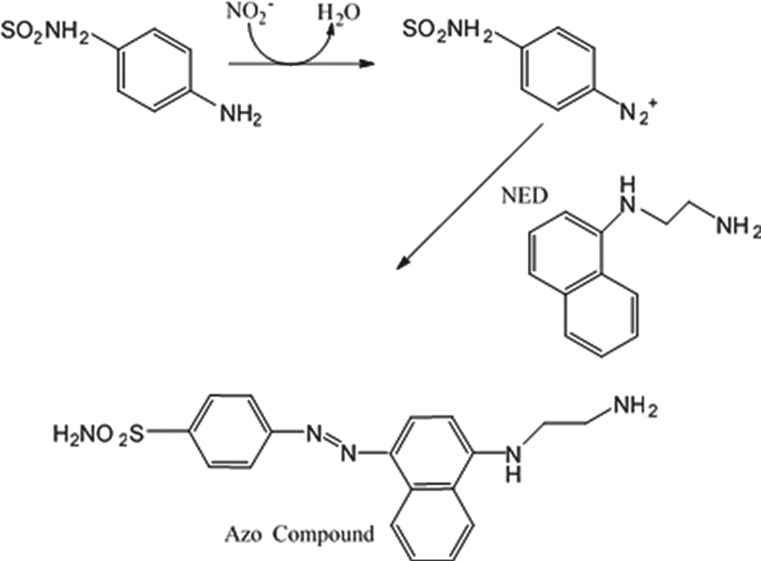
A schematic showing the formation of the azo compound upon the interaction of nitrite with the Griess reagent.

The colorimetric assay for the deamination of the nitrite concentration was prepared by adding different nitrite concentrations to the colourless nitrite assay reagents, which resulted in a colour change based on the Griess assay [44,45]. The Griess assay is one of the most well-known methods for the colorimetric detection of nitrite and has been used since 1879. A typical Griess assay relies on the formation of diazonium salt due to the reaction of sulfanilamide with nitrite under acidic conditions. The formed diazonium salt creates a coloured azo dye upon reacting with NED. Figure 3 shows a scheme of the colorimetric detection of nitrite using the Griess reaction. Like all other colorimetric assays, a spectrophotometer is needed for analysis which makes it unsuitable for on-site detection. Hence, there is a need to develop a new POC analysis tool for colorimetric assays [46,47]. The proposed device allows rapid detection of pH values and nitrite concentrations, and also a replication of the colorimetric assays in a single unit.

A UV–vis, Evolution 60 s (Thermo Fischer Scientific, Madison, WI, USA) was used to determine the absorption of nitrite. The same experimental conditions were used for the absorption determination and during tests of the developed device. Water was used as a blank to generate the baseline. All samples were measured at 540 nm, and a zero absorbance was set before any measurements were taken of the nitrite concentrations. Samples were prepared in cuvettes and experiments were conducted at room temperature.

### 2.4. Device calibration

Aqueous solutions of phosphate buffers of various known pH values and solutions of different nitrite concentration were made to calibrate the device for pH measurement. Sodium phosphate monobasic monohydrate and sodium phosphate dibasic were mixed to prepare phosphate buffer solutions with varying pH values. All aqueous solutions were prepared using DI water. An Oakton pH meter (Oakton Instruments, pH 510 series) was calibrated against three standard buffer solutions with three different pH values (4.0, 7.0 and 10) and was then used for the pH determination.

The number of reference points to be stored was the primary parameter to be determined for calibration. Several images of each sample were taken for calibration. The images were taken using the integrated camera at a fixed distance of 1 cm, at room temperature, and under fixed and stable lighting conditions. All images were taken and processed within 1 s, and the calibration data points were stored in the device memory.

### 2.5. Colorimetric testing method

The portable device shown in figure 1 records an image of the coloured sample; the image information is then processed and converted to the corresponding pH value or nitrite concentration by the developed detection algorithm. The values are displayed on the touchscreen. To perform the colorimetric assay for the determination of pH, 1 ml of a phosphate buffer solution of known pH value (prepared for calibration) and different solutions with unknown values (for data validation) were mixed with 250 µl of bromothymol blue and 50 µl of methyl red. All reagents were mixed thoroughly. A period of 5 s was needed to produce the colour change in a 1 ml cuvette. To perform the nitrite colorimetric test, 1 ml of each sodium nitrite concentration was transferred to a 1 ml cuvette. Then, 50 µl of 1% sulfanilamide solution was added to the sample, and the cuvette was capped and mixed by inverting three times. Then, 50 µl of the 0.02% NED solution was added, and the cuvette was inverted three times again. The cuvette was left at room temperature for 5 min to develop colour and was then inserted into the portable device for measuring.

Once all the reagents were allowed to react and had produced a colour change, a picture of the coloured sample was taken and processed. The detection process was carried out under controlled lighting conditions at room temperature and using the exact conditions and steps followed for generating the calibration curve. The HSV coordinates were extracted from the coloured image, analysed and converted to the corresponding pH value or nitrite concentration by comparing the analyte data with the calibration data using the developed algorithm.

### 2.6. Software

**Figure 4.**
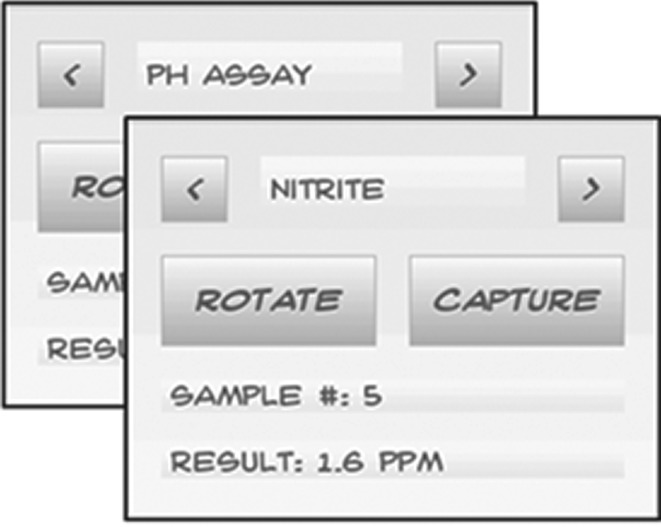
The prototype GUI designed using the PyGameUI library for Python.

Software for the colorimetric device was written in Python. Support libraries used were OpenCV for image processing, and Pygame and PygameUI for developing the custom graphical user interface (GUI), allowing the selection of a particular, or all five cuvettes in the carousel. After capturing the images, the nitrite concentration or the pH value of the sample is displayed on the screen (figure 4).

### 2.7. Image processing

**Figure 5.**
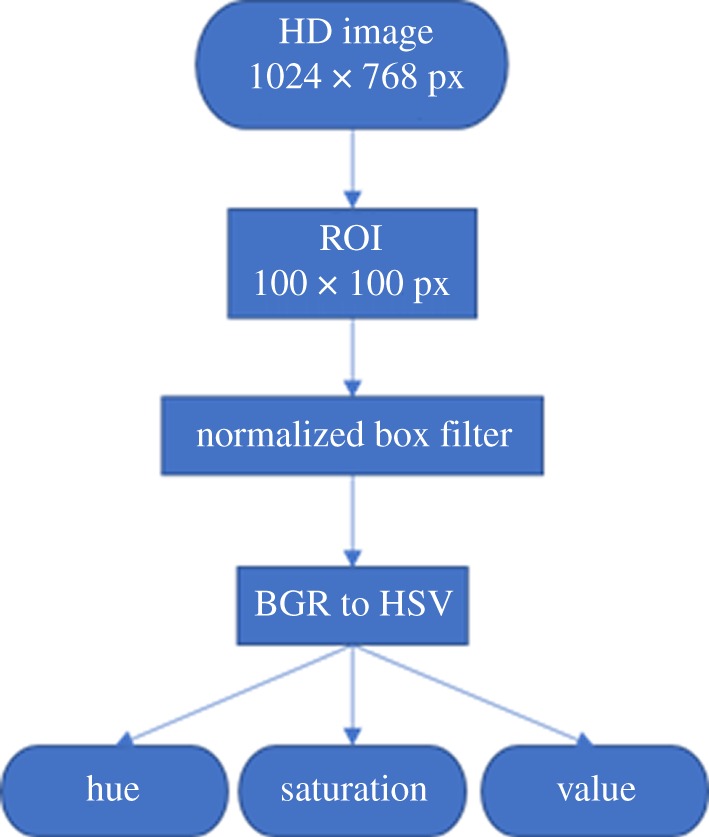
Image processing pipeline performed using OpenCV.

**Figure 6.**
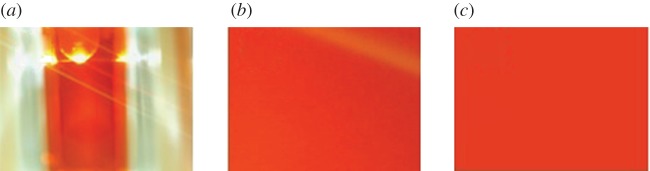
Images in various stages of processing using the OpenCV library for Python. (*a*) Original image (1024 × 768 pixels), (*b*) ROI (100 × 100 pixels) and (*c*) after application of 200 × 200-pixel box filter (100 × 100 pixels).

The OpenCV library was used to perform image analysis [48] (figure 5). The original image of each cuvette was captured at the full resolution of 1024 × 768 pixels (figure 6*a*). Then, a region of interest (ROI) at the size of 100 × 100 pixels was obtained around the centre of the cuvette (figure 6*b*). A box filter smoothening function (box blur) was applied to the ROI to obtain a uniform representation of the average pixel colour value. The normalized box smoothening computes the value of each pixel as the mean of its kernel neighbours. The average pixel value of the ROI is computed using 200 × 200 pixels which are double the size of the ROI in both horizontal and vertical directions (figure 6*c*). In this way, the image-processing algorithm yields consistent results even with slight inconsistencies in the cuvette location or external lighting. The ROI was converted from the red, green, blue (RGB) colour space to the HSV colour space to achieve a linear relationship between the colour change and colour components.

## 3. Results

### 3.1. pH detection

#### 3.1.1. Calibration curve

**Figure 7.**
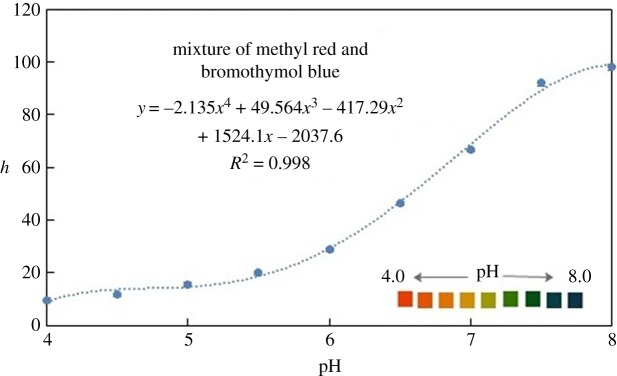
The hue values as a function of pH values (ranging from 4 to 8) and the fitted polynomial curve.

For the determination of pH values, the colorimetric test for each pH value was performed three times under the same conditions using a mixture of bromothymol blue and methyl red. The period needed for the detection was 5 s from the time the sample was inserted into the portable device until the image was captured. The response of the mixed indicators was studied using phosphate buffer solutions of various pH values ranging from 4 to 8. Figure 7 shows the change in the hue coordinate for the mixed indicator, with a value ranging between 5 and 110. This wide range of values indicates that the hue coordinate can provide a significant measurement with sufficient resolution for the pH value of each indicator solution. A fourth order polynomial curve was fitted to the experimental data, enabling determination of the pH value of an unknown solution from the hue value obtained using the device.

### 3.2. Nitrite detection

#### 3.2.1. Detection limit and calibration curve

**Figure 8.**
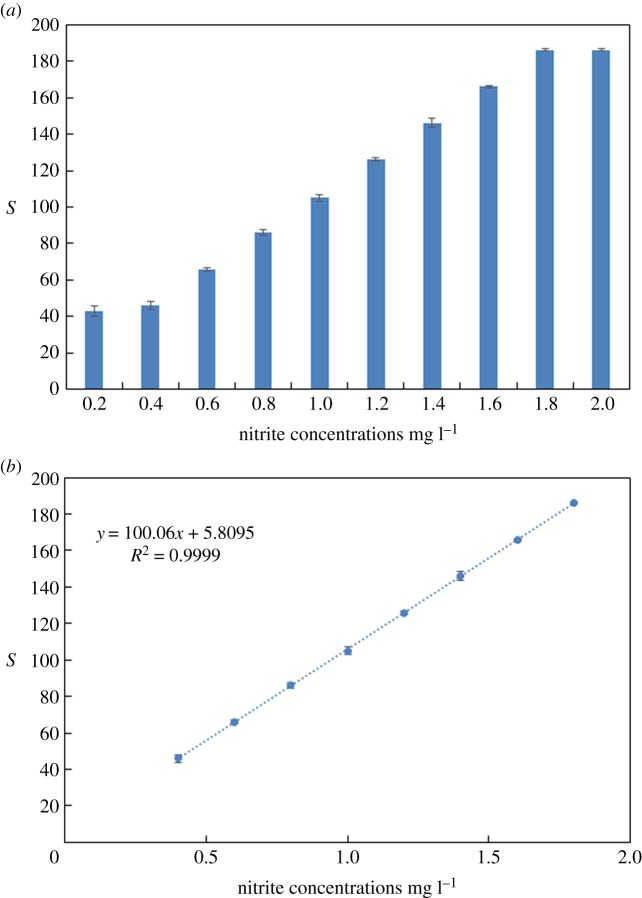
(*a*) The sensor response to different nitrite concentrations in the sample and (*b*) nitrite concentration calibration curve (line fitted to the experimental *S* values obtained for different nitrite concentrations using Griess reagents).

For the determination of the nitrite concentrations, the colorimetric test was repeated three times under the same conditions mentioned in the previous sections. The formed azo dye from the reaction between the nitrite and Griess reagents results in a colour change, from colourless to pink, then to red as the nitrite concentration increased in the samples. The results also show that the (*S*) value of the image remains constant above 1.8 mg l^−1^ of nitrite concentration in the sample (figure 8*a*).

The time needed for the detection was 5 min, from when the sample was mixed until the image was captured. Owing to the colourless nature of the Griess solution used for the detection of the nitrite concentration, the saturation coordinate (*S*) proved to be the most accurate and reliable coordinate for measuring the colorimetric change, as it is proportional to the nitrite concentration in the sample. The sensor response (*S* value) from different nitrite concentrations and the generated calibration curve are shown in figure 8*a* and *b*, respectively. In this case, the calibration curve is a line, and the correlation coefficient *R*^2^ was 0.9999. The minimum detection limit achieved for nitrite was 0.2 mg l^−1^ (figure 8*a*), which is below the acceptable range for drinking water [49], which is 1 and 3 mg l^−1^ according to the US Environmental Protection Agency [50] and World Health Organization (WHO) [51], respectively. The experimental error bars in figures 7 and 8 were too small to be seen, confirming the high precision of the data generated by the portable device.

### 3.3. Data validation

Additional experiments were carried out to examine the repeatability, accuracy and reliability of the measurements using the portable device. For the pH data validation, five phosphate buffer solutions, with different pH values, were prepared. The mixture of pH indicators (methyl red and bromothymol blue) was used for the data validation. Phosphate buffer solution (1 ml) was mixed with 50 µl of methyl red and 250 µl of bromothymol blue to test the pH value. All tests were completed in a 1 ml cuvette. Reagents were mixed thoroughly and placed in the device for 5 s, for the colour change to occur. All tests were conducted three times. To perform the nitrite colorimetric test, the portable device was tested against four random samples with different nitrite concentrations (0.5, 1.0, 1.5 and 2.0 mg l^−1^).

**Figure 9.**
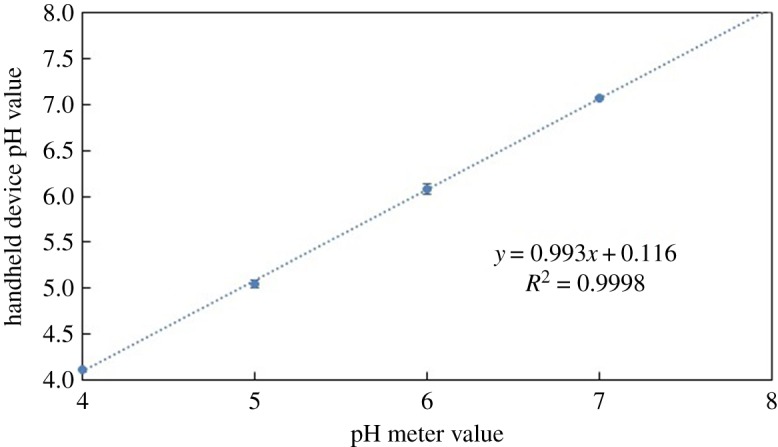
A linear relationship between the pH values obtained using the pH meter and those obtained using the portable device.

**Figure 10.**
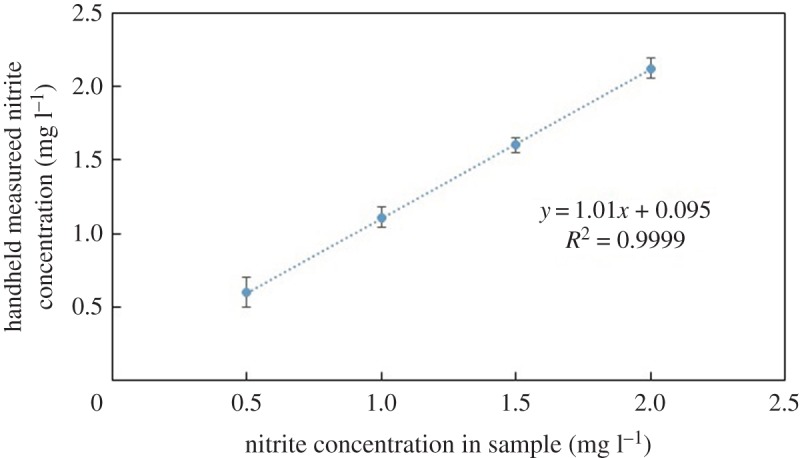
A linear relationship between the concentrations of the prepared nitrite solutions and those obtained using the portable device.

All reagents were allowed to react and produce a colour change before images of the samples were taken and processed by the device. The detection process occurred under the same conditions as those performed for the calibration process. Tables 2 and 3 summarize a comparison between the data obtained using traditional analyses and those obtained by the portable device for the pH values and nitrite concentrations, respectively. In general, the results are in good agreement, with maximum errors occurring at the lowest pH values and nitrite concentrations. To have a better presentation of these comparisons, the values listed in tables 2 and 3 are graphed using a unity line (figures 9 and 10). The data generated by the device have a mean standard deviation value of 0.03 and 0.073, and correlation factor, *R*^2^, of 0.998 and 0.999 for the pH values and nitrite concentrations, respectively. These results confirm that the portable device is accurate and reliable for the rapid detection of pH and nitrites.

**Table 2.** Comparison between the pH readings obtained using the pH meter and the portable device.

pH meter value	portable device measured pH value
4	4.1
5	5.04
6	6.08
7	7.07
8	8.05

**Table 3.** Comparison between the results of the nitrite solutions prepared against those obtained by the portable device.

nitrite concentration in sample (ppm)	the portable device measured nitrite concentration (ppm)
0.5	0.6
1.0	1.11
1.5	1.6
2.0	2.12

In figures 8 and 10, the detection values of nitrite at a concentration of 0 mg l^−1^ were not measured due to the colourless nature of the Griess solution used for the detection of the nitrite concentration.

### 3.4. Nitrite measurement comparison

**Figure 11.**
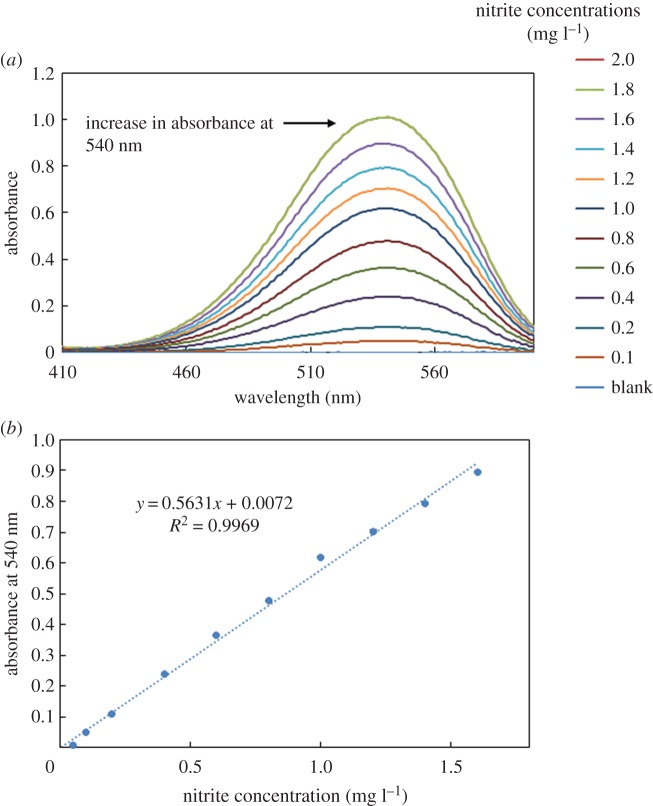
UV–vis measurements for nitrite concentrations (*a*) UV–vis spectrum for the determination of different nitrite concentration and (*b*) nitrite concentration calibration curve (line fitted to the experimental absorbance values at 540 nm obtained for different nitrite concentrations using Griess reagents).

A comparison study was carried out between the produced data for nitrite detection by the developed device and traditional UV–vis spectroscopy. Figure 11*a*,*b* shows a full spectrum of UV absorption and the produced calibration curve of different nitrite concentrations, respectively.

Table 4 shows a comparison between the developed colorimetric device and the traditional UV–vis for nitrite detection.

**Table 4.** Comparison of the data obtained by UV–vis spectroscopy, the developed device and other methods reported in the literature.

method	linear detection range (mg l^−1^)	limit of detection (mg l^−1^)	portability	ref.
UV–vis	0.1–1.6	0.1	no	this work
HPLC–UV	0.1–100.0	0.05	no	[52]
Spectrofluorometric	0.005–0.500	2.5 × 10 ^−3^	no	[53]
Spectrophotometric ion-pair HPLC	0.01–0.1	0.1	no	[54]
our device	0.4–1.6	0.2	yes	this work

The comparison of the results and portability in table 4 shows that the developed portable colorimetric device possesses a broad linear range and a low detection limit when compared with other methods reported in the literature for the detection of nitrite. Furthermore, the device is simple to use and does not require trained personnel to operate it. It should be noted that the price to manufacture our device is significantly lower than that of the other methods, approximately four to five times cheaper, which is a significant advantage for the overall proliferation of the technology. Also, the device showed another significant advantage over the UV–vis spectroscopy and HPLC, portability, which makes the device more suitable for online and remote-area detection.

## 4. Discussion

The use of smartphones for sensing colour change is often subject to different light conditions, resulting in a high possibility of interference from ambient light. This significantly limits the use of these devices in sensing technologies and for POC applications. Hence, there is a need to overcome this obstacle using (i) a stable and reliable external source of light for all measurements and (ii) a designated means of keeping the distance between the samples and camera constant [38,39]. Also, spectrophotometric absorption is one of the widely used technologies for detection of colorimetric assays. However, this instrument is bulky and expensive, and needs trained personnel to run it, which makes it inadequate for POC applications [55]. As a result, developing a versatile, low-cost, rapid and portable colorimetric device is important and needed for on-site detection.

This work demonstrates the development of a low-cost and versatile portable colorimetric detection device for on-site detection and potentially for POC diagnostics. Three-dimensional printing technology enabled fast prototyping for this project. The three-dimensional-printed design was customized to (i) accommodate the rotating carousel mounted on a motor to manipulate five separate cuvettes around the camera for multiple sample analysis; (ii) contain an LED as the external light source that controls the light conditions during image capture; (iii) rapidly analyse the colour change in 8-megapixel resolution images and (iv) contain onboard electronics that provide fast and robust detection of the HSV coordinates of the captured images. While using higher quality components, specifically a higher resolution camera, may slightly increase precision, upgrading hardware should not significantly change data acquired. Additionally, the developed device is battery-powered, wirelessly connectable and equipped with a Raspberry Pi computer for data processing, storage and exchange to make it suitable for POC diagnostics. It could be envisioned that outputs for data may be interfaced with a smartphone in the future, to further increase accessibility without comprising accuracy or precision [56].

## 5. Conclusion

The developed colorimetric device shows several advantages compared with the traditional method for colorimetric detection, using UV–vis spectroscopy. First, small and inexpensive off-the-shelf components were employed in the construction of the device including (i) Raspberry Pi computer; (ii) Raspberry Pi Camera Module and (iii) 2.8′′ PiTFT touchscreen. As a result, a new portable device is developed that not only has a high degree of versatility but also provides a new cost-effective method for colorimetric detection compared with the traditional bulky, expensive instruments. Second, a novel algorithm was developed to analyse the image taken that decreases the influence of ambient light and varying positions of the sample in front of the camera during the image-capturing process. Unlike other image-based devices used for detection of colorimetric assays, the use of an integrated camera in the proposed portable device minimizes human error, including hand motion of the user. Also, establishing a fixed distance between the sample and the camera has resulted in less interference from ambient light, high repeatability, sensitivity and reliability. All these advantages have made the device suitable for personal use and POC testing in different environmental light conditions. This low-cost and user-friendly analytical tool for in-field and POC diagnostics can perform multiple detections with no need for external processing tools, which decreases the analysis time, reduces the cost and simplifies the procedure for untrained personnel.

Third, the efficacy of the device with respect to two different applications demonstrated the versatility and ease of use of the device for all colorimetric assays. Specifically, a simple calibration process can quickly prepare the device for other applications at no additional cost. For example, the colorimetric analysis technique and the algorithm developed in this work are not limited to water analysis and can be used for urinalysis and other colorimetric assays such as colloidal gold nanoparticles for environmental and medical applications [57], other hazardous species and ELISA assay [9]. In future work, the developed device will be tested with real samples under different physical and environmental conditions including interference with other substances in the sample. This step will demonstrate and verify the reliability of the device and its ease of handling and operation by users.

## Data accessibility

All data are available at the Dryad temporary review link (http://datadryad.org/resource/doi:10.5061/dryad.pj475) [58].
